# Successful Surgical Sperm Extraction in a Patient With Cystinosis

**DOI:** 10.7759/cureus.102787

**Published:** 2026-02-01

**Authors:** Moshe Wald

**Affiliations:** 1 Urology, University of Iowa Hospitals and Clinics, Iowa City, USA

**Keywords:** azoospermia, cystinosis, in-vitro fertilization, surgical sperm retrieval, testicular sperm extraction

## Abstract

Cystinosis is a genetic lysosomal storage disease with an autosomal recessive inheritance pattern. The mutation causing this disease occurs in the *CTNS* gene, which is located on chromosome 17 and codes for the lysosomal cystine transporter cystinosin. Cystinosis is characterized by intracellular accumulation of cystine in various organs and tissues, which may lead to impairment of organ function. Kidney injury is characterized by a progressive decline in glomerular filtration rate, leading to end-stage kidney disease if not treated. Other manifestations of cystinosis include ocular abnormalities, hepatomegaly, hypothyroidism, muscle weakness, and growth retardation. Male patients with cystinosis typically have azoospermia, and current data regarding the existence of spermatogenesis and the possibility of surgical sperm retrieval in these patients are limited.

This article presents a case of a male patient in his thirties with cystinosis and azoospermia who presented for evaluation and treatment of infertility. His serum testosterone was normal, but gonadotropin levels were markedly elevated. Y chromosome micro-deletion assay was negative, and the karyotype was normal. Testicular sperm extraction revealed the presence of viable mature sperm that were successfully harvested and cryopreserved. The patient’s spouse conceived through an in vitro fertilization cycle that utilized the patient’s surgically retrieved sperm and subsequently delivered a healthy baby boy.

This case report provides novel information on post-thaw quality measures of sperm that were surgically retrieved from a patient with cystinosis, as well as on outcomes of in vitro fertilization that utilized such sperm. The post-thaw quality of sperm is associated with its utility for in vitro fertilization and thus adds to the limited data available thus far regarding the feasibility of surgical sperm retrieval in patients with this disease.

## Introduction

Cystinosis is a genetic lysosomal disease with an autosomal recessive inheritance pattern. It is characterized by the intracellular accumulation of cystine in various organs and tissues, which in turn may lead to disruption of organ function. The cystinosis gene (*CTNS*) is located on the short arm of chromosome 17 and encodes for cystinosin, a lysosomal membrane protein responsible for exporting cystine from lysosomes [1]. Three forms of cystinosis have been described, including infantile, late-onset (juvenile), and adult (benign) forms. Infantile or nephropathic cystinosis is the most common form of cystinosis and has been estimated to affect one of every 100,000-200,000 children [2,3].

Cystine, the product of protein breakdown within the lysosomes of cells, is typically transported through the lysosomal membrane to the cytosol, where it is transformed to cysteine for subsequent use. In cystinosis, a defect in the *CTNS* gene interrupts the production of cystinosin, which in turn results in the accumulation of cystine in the lysosomes. Cystine is poorly soluble and forms crystals as its concentration increases. The mechanism by which cystine accumulation causes cellular dysfunction is not clear [4], but multiple organs may be affected to varying degrees in cystinosis, including the kidneys, brain, skeletal muscles, eyes, bone, and the reproductive system.

Male patients with cystinosis usually have azoospermia, commonly accompanied by low serum testosterone levels and high follicle-stimulating hormone (FSH) and luteinizing hormone (LH) levels [5,6]. The latter hormonal abnormalities may compromise pubertal development and suggest a non-obstructive nature of the azoospermia in these patients. Only limited data are available regarding the status of spermatogenesis in patients with cystinosis. Two small studies that assessed fertility status in male patients with cystinosis reported a total of only three patients who underwent testicular biopsies, in which spermatogenesis was present [5,7]. However, these studies did not include information on whether in vitro fertilization was subsequently performed. Gathering more information on the status of spermatogenesis in patients with cystinosis is of clinical significance to assess the potential utility of surgical sperm retrieval for in vitro fertilization as a fertility treatment modality in these patients. The objective of this case report is to provide information regarding the quality and clinical utilization of surgically retrieved sperm in a patient with cystinosis.

## Case presentation

A 37-year-old male was referred to our male infertility clinic for evaluation of infertility and azoospermia. The patient and his spouse had attempted to achieve pregnancy for seven years prior to his referral and were unsuccessful.

The patient reported a history of nephropathic cystinosis, hypertension, and hypothyroidism. He was first diagnosed with nephropathic cystinosis at 11 months of age during a workup for growth failure. He underwent kidney transplantation eight years ago. His serum creatinine was 0.73 mg/dL (0.67-1.17 mg/dL) at the time of presentation to our clinic. His medications included amlodipine, levothyroxine, lisinopril, mycophenolate mofetil, tacrolimus, cysteamine bitartrate, and omeprazole. He denied any problems with erections or ejaculation. His spouse is a female in her thirties who reported regular menstrual cycles and had not been pregnant previously.

Genital examination revealed a normal circumcised penis. The testes were bilaterally descended, of normal size, without palpable masses or tenderness. The vasa were palpable, and no varicocele was appreciated.

The patient had undergone a semen analysis five months prior to presentation, which demonstrated normal-volume azoospermia. A panel of reproductive hormones obtained six months prior to presentation revealed a serum testosterone level of 580 ng/mL (249-836 ng/mL), FSH of 45.3 milli international units/mL (1.5-12.4 milli international units/mL), and LH of 19.7 milli international units/mL (1.7-8.6 milli international units/mL). The marked elevation in serum FSH and LH levels suggested primary testicular dysfunction and a non-obstructive etiology of the patient’s azoospermia. A second semen analysis performed one month after the initial evaluation in our clinic confirmed normal-volume azoospermia. As part of the complete azoospermia workup, the patient also underwent karyotype analysis, which was normal at 46, XY, and Y chromosome microdeletion testing, which was negative, with no deletions detected in the AZFa, AZFb, or AZFc regions (Table 1).

**Table 1:** Summary of laboratory tests LH: luteinizing hormone, FSH: follicle-stimulating hormone.

	Serum creatinine (mg/dL)	Serum total testosterone (nanogram/mL)	Serum LH (milli international units/mL)	Serum FSH (milli international units/mL)	Karyotype	Y chromosome micro-deletion assay	Post-thaw sperm viability (%)
Patient results	0.73	580	19.7	45.3	46,XY	Negative	39
Reference range	0.67-1.17	249-836	1.7-8.6	1.5-12.4	46,XY	Negative	≥58

The patient’s medications were unlikely to be the cause of his azoospermia. Additionally, the fact that his kidney transplantation was performed eight years ago, along with his normal serum creatinine level at presentation to our clinic, suggested that his azoospermia was not related to renal issues.

**Figure 1:**
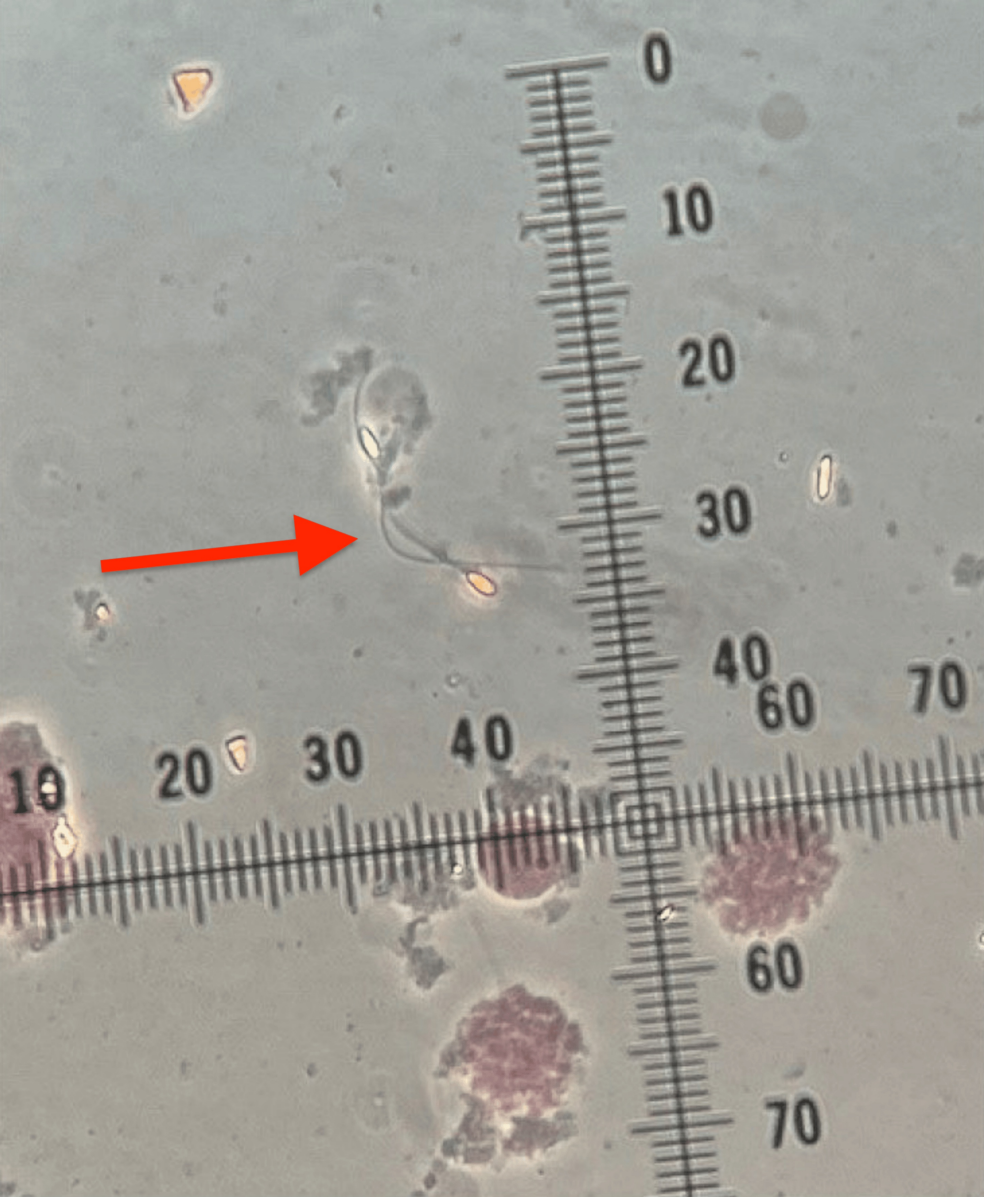
Spermatozoa identified in testicular tissue sample processed and analyzed by andrologist during testicular sperm extraction surgery A small portion of testicular tissue has been carefully separated into several tiny pieces, each of which was placed in a Petri dish containing gamete media that had the following composition: Modified HTF (human tubal fluid) medium with HEPES (4-(2-hydroxyethyl)-1-piperazineethanesulfonic acid, a buffering agent that is widely used in cell cultures) (Irvine Scientific, Catalog #90126) with Quinn's Advantage Serum Protein (7.5%) (Cooper Surgical, Catalog #3011). Each piece of testicular tissue then underwent careful microscopic examination by an andrologist in the operating room to assess for the presence of spermatozoa, which were identified and shown by the arrow in this figure.

Following completion of his evaluation, the patient underwent testicular sperm extraction (TESE), which was performed as an outpatient procedure under general anesthesia. The right testicle was delivered through a short midline scrotal incision. The tunica vaginalis was opened along its anterior aspect, exposing the tunica albuginea. A short incision was made in the tunica albuginea. The testicular parenchyma appeared somewhat more condensed. Several small samples of testicular tissue were sharply harvested and handed to an andrologist for examination in the operating room, which revealed the presence of mature sperm (Figure 1). 

The tunica albuginea was closed with a 5-0 Prolene (polypropylene) suture in a running fashion. The tunica vaginalis was closed with a running 4-0 Vicryl (polyglactin 910) stitch, and the right testicle was returned to its anatomical position. Given the identification of sperm in the specimens from the right testicle, surgery was limited to this side. The dartos layer and subsequently the skin were then closed in separate layers. The patient tolerated the procedure well, and there were no intraoperative or immediate postoperative complications. Right testicular tissue samples were taken to the Reproductive Testing Laboratory at our institution for further analysis and cryopreservation. Eight biopsies were taken during right TESE and cryopreserved into seven vials. Post-thaw viability was determined by eosin dye staining (200 sperm observed) and was found to be 39%. The Reproductive Testing Laboratory at our institution estimated that the cryopreserved sperm would allow for up to seven in vitro fertilization cycles.

At follow-up one month after surgery, the patient was doing well, with no pain, and had returned to daily activities and work. The scrotal incision was healing well. His wife conceived through an in vitro fertilization cycle that utilized the patient’s surgically retrieved sperm and subsequently delivered a healthy baby boy.

## Discussion

The patient presented in this article has a history of nephropathic cystinosis, which is the most common form of cystinosis, characterized by both renal and extrarenal symptoms that present after birth [8]. Extrarenal manifestations of cystinosis include ocular abnormalities, hepatomegaly, hypothyroidism, muscle weakness, and growth retardation. Male patients with cystinosis typically have azoospermia. The patient presented in this case report sustained severe renal failure related to cystinosis and underwent kidney transplantation eight years ago. His extrarenal manifestations of cystinosis were limited to azoospermia.

Several possible mechanisms for cellular dysfunction in cystinosis have been suggested. These include oxidative stress and apoptosis related to depletion of the glutathione cell pool by cystine [9,10], as well as impaired chaperone-mediated autophagy [11]. Additionally, overexpression of intracellular clusterin was observed in patients with cystinosis, which may contribute to cell stress, injury, and apoptosis [12]. Cystinosin deficiency could also lead to reduced cell adhesion and hypermotility [13].

Male patients with cystinosis typically have azoospermia, but spermatozoa were reported to be present in the semen of three out of fifteen men with cystinosis [7]. While the impairment of testicular function in men with cystinosis has been attributed to lysosomal cystine overload in both Sertoli and Leydig cells, a component of obstruction in certain parts of the male reproductive tract has been suggested to contribute to impaired semen quality in these patients. Findings supporting a possible obstructive mechanism include dilation of the rete testis on ultrasound, as well as reduced semen volume and fructose levels that were reported in some men with cystinosis [7].

In cases of azoospermia without a correctable cause (for example, obstruction along the male reproductive tract that is amenable to surgical repair, such as prior vasectomy or obstruction of the ejaculatory ducts), fertility treatment typically centers on surgical sperm retrieval, combined with in vitro fertilization. The latter can be either coordinated with the surgery for sperm retrieval (in which case fresh sperm is utilized), or can be performed later, using sperm that has been cryopreserved. Only limited data are currently available regarding the status of spermatogenesis and the feasibility of surgical sperm retrieval in patients with cystinosis. Only three cystinosis patients who underwent testicular biopsies were reported in two small studies that assessed fertility status in this disease. Spermatogenesis was present in these patients, and sperm was cryopreserved in two of them [5,7]. These studies did not include information on whether in vitro fertilization was subsequently performed.

Our case report adds to the limited data regarding fertility treatment in male patients with cystinosis, suggesting that testicular sperm extraction may be considered as a treatment option in this type of azoospermia, giving hope to cystinosis patients seeking to have biological children. Furthermore, to our knowledge, this article is the first to provide data regarding the post-thaw quality of cryopreserved sperm that has been surgically retrieved from a patient with cystinosis. The post-thaw sperm viability reported in this article is encouraging and, along with the success of the patient’s wife’s in vitro fertilization treatment, further supports the combination of testicular sperm extraction and in vitro fertilization as a possible approach for the fertility treatment of couples when the male partner has cystinosis and azoospermia. However, this is a single case report, and further information is needed regarding both the feasibility of surgical sperm retrieval and the subsequent clinical use of such sperm in patients with cystinosis.

## Conclusions

This case report presents successful surgical sperm retrieval in a patient with cystinosis and azoospermia and includes reassuring data regarding the quality of the harvested sperm. The latter adds to currently limited information about testicular biopsy findings in men with cystinosis and supports testicular sperm extraction as a feasible fertility treatment option in these patients. Additional studies, including outcomes of IVF utilizing surgically retrieved sperm from cystinosis patients, would be needed to further investigate TESE/IVF as a fertility treatment modality for couples in which the male partner has cystinosis and azoospermia.
